# Feasibility and surgical outcomes of video‐assisted thoracoscopic pulmonary resection in patients with advanced‐stage lung cancer after neoadjuvant chemoradiotherapy

**DOI:** 10.1111/1759-7714.13074

**Published:** 2019-04-13

**Authors:** Jee Won Suh, Seong Yong Park, Chang Young Lee, Jin Gu Lee, Dae Joon Kim, Hyo Chae Paik, Kyoung Young Chung

**Affiliations:** ^1^ Department of Thoracic and Cardiovascular Surgery, Severance Hospital Yonsei University College of Medicine Seoul South Korea

**Keywords:** Chemoradiotherapy, lung neoplasm, neoadjuvant therapy, thoracotomy, video‐assisted

## Abstract

Video‐assisted thoracoscopic surgery (VATS) is regarded as the standard treatment for lung cancer. However, the feasibility and safety of VATS for lung cancer after neoadjuvant chemoradiotherapy (CRT) is unclear. This study evaluated the feasibility and safety of VATS in patients who had received neoadjuvant CRT.

**Methods:**

Between January 2008 and December 2017, 85 patients who were administered neoadjuvant CRT and underwent anatomic lung resection were enrolled. Fifty‐nine patients underwent open thoracotomy and 26 patients underwent VATS. The clinical characteristics and perioperative outcomes were reviewed.

**Results:**

In six of the initial 32 patients who underwent VATS, the procedure was converted to thoracotomy. Adjacent structural invasion (33.9% vs. 11.5%; *P* = 0.037) and combined resection (16.9% vs. 0%; *P* = 0.025) were higher in the open group than in the VATS group. Surgical duration was higher in the open group than in the VATS group (203.86 ± 65.97 vs. 173.27 ± 59.87 minutes; *P* = 0.046). With regard to postoperative outcomes, the length of the hospital stay was longer in the open group compared to the VATS group (14.46 ± 16.94 vs. 8.62 ± 4.72 days; *P* = 0.017). There was no significant difference in the three‐year disease‐free survival (69.3% vs. 67.9%; *P* = 0.879) or overall survival rates (76.6% vs. 61.9%; *P* = 0.516).

**Conclusion:**

In selected patients, VATS pulmonary resection after neoadjuvant CRT showed results comparable to that of thoracotomy in terms of postoperative outcomes, operative morbidities, and survival rate.

## Introduction

Since the 1990s, video‐assisted thoracoscopic surgery (VATS) has become a popular method for performing pulmonary resection for lung cancer and is now regarded as the standard treatment for early‐stage non‐small cell lung cancer (NSCLC).^1^, ^2^ VATS lobectomy offers more advantages than thoracotomy lobectomy in terms of short‐term postoperative outcomes, including less postoperative pain, faster postoperative recovery, greater preservation of pulmonary function, and lower perioperative morbidity.^3^ Moreover, VATS lobectomy is equivalent to thoracotomy in terms of oncologic outcomes, including survival and recurrence rates.^4^, ^5^

Recent studies have suggested that surgical resection following neoadjuvant chemotherapy for patients with locally advanced NSCLC can significantly improve the complete surgical resection (R0) rate and long‐term survival.^2^, ^6^, ^7^ However, the feasibility and clinical efficacy of performing VATS pulmonary resection after neoadjuvant CRT remains controversial.^3^, ^8^ The technical difficulties associated with VATS pulmonary resection following neoadjuvant therapy are related to the formation of adhesions and increased tissue fragility, which affect patient recovery.^8^ Therefore, the objective of this study was to evaluate the feasibility and safety of performing anatomical lung resection under VATS in patients with advanced‐stage lung cancer who had received neoadjuvant CRT.

## Methods

**Figure 1:**
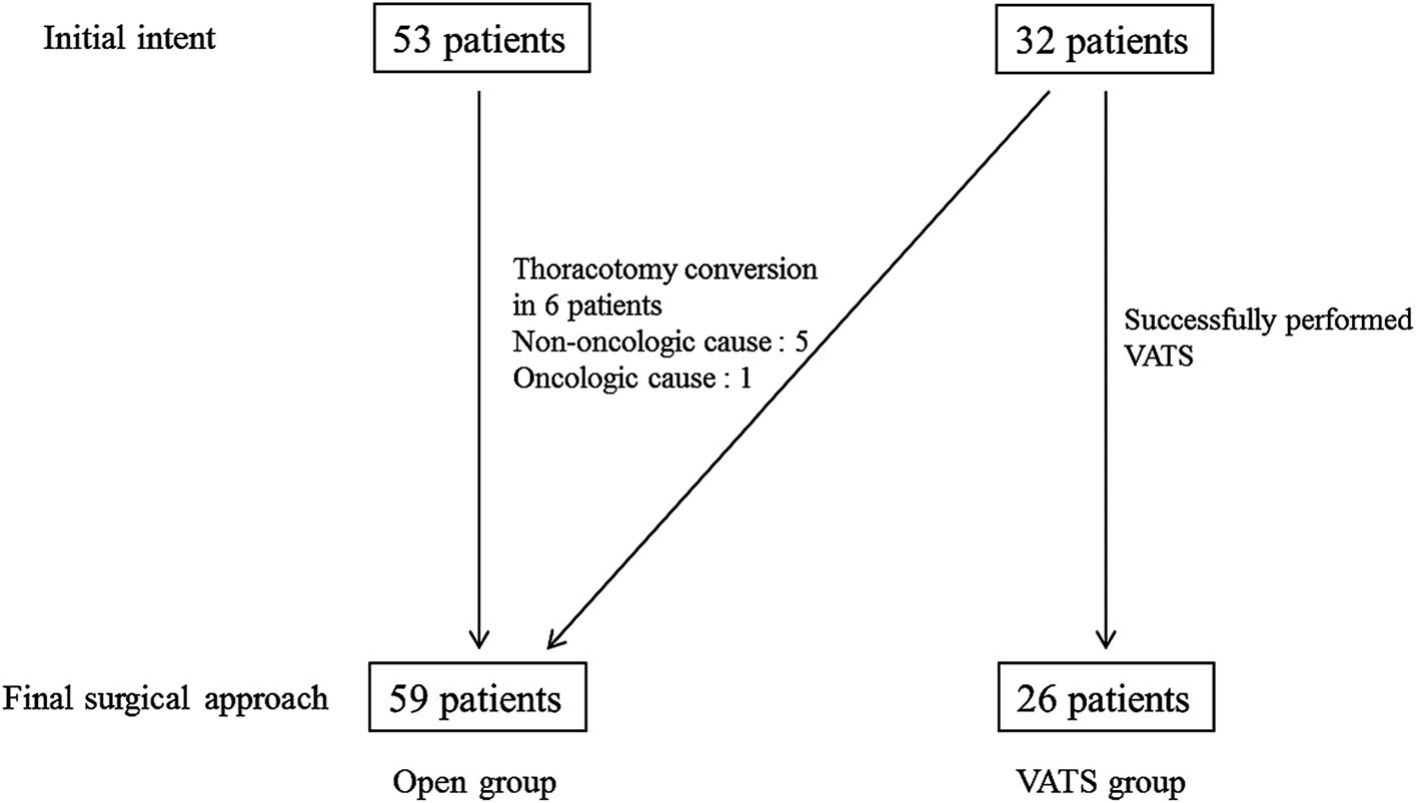
Patient enrollment. VATS, video‐assisted thoracoscopic surgery.

The Institutional Review Board of Severance Hospital approved this retrospective study (4‐2018‐0414). Patients who underwent surgical resection for lung cancer following neoadjuvant CRT at our institution from January 2008 to December 2017 were enrolled. Patients who underwent non‐anatomical and sublobar resection were excluded from this analysis. A total of 85 patients were enrolled in the study: 59 patients (69.4%) who underwent thoracotomy (including 6 patients in whom initial VATS was converted to thoracotomy) were categorized as the Open group; and 26 patients (30.6%) who successfully underwent VATS were categorized as the VATS group (Fig 1). All enrolled patients had received cisplatin‐based chemotherapy and radiation therapy prior to surgery.

### Surgical methods

Anatomical resection (lobectomy, bilobectomy, or pneumonectomy) and mediastinal lymph node dissection were performed in addition to en bloc removal of any of the involved adjacent structures, including the large vessels and chest wall. In the open group, surgical resection under thoracotomy was performed with an incision placed at the fifth intercostal space (ICS); an anterolateral incision or posterolateral incision was also used. In the VATS group, surgical resection was performed using four incisions. Two 12 mm inferior port incisions were positioned at the level of the seventh ICS crossing at the anterior axillary line and the eighth ICS crossing at the posterior axillary line. An additional posterior 5 mm port incision was positioned inferior to the scapular tip. A 4 cm incision was created at the level of the fourth ICS between the latissimus dorsi and pectoralis major muscles for use as a utility port without rib spreading. When VATS was converted to open thoracotomy, the utility incision was extended to the posterior 5 mm port incision.

### Operative outcomes and follow‐up

Follow‐up and clinical outcome data were collected from the patients’ medical records. Analytical data included: age; gender; smoking history; performance status (based on Eastern Cooperative Oncology Group criteria); pulmonary function test results; tumor invasion of adjacent structures (chest wall, mediastinum, and large vessels); and tumor size, location, and histology. Operative outcomes included resection type, the need for combined procedures, the presence of pleural adhesions, surgical duration, and estimated blood loss. Postoperative outcomes included admission to the intensive care unit, duration of chest tube drainage, length of hospital stay, pathologic stage, R0 resection rate, number of dissected mediastinal lymph nodes (LNs), complications, operative mortality, and recurrence. Complications included postoperative bleeding, prolonged chest tube drainage, pneumonia/acute respiratory distress syndrome, and bronchopleural fistula (BPF). Locoregional recurrence was defined as intrathoracic disease in the lung or nodal recurrence in the hilum or mediastinum. Distant metastasis was defined as extrathoracic metastasis to one or more organs.

### Statistical analysis

We used descriptive statistics to calculate the percentage, mean, standard deviation, and median for the selected demographic and clinical parameters. Comparisons between the groups were analyzed using Student's *t*‐test for continuous variables and the chi‐square test for categorical variables. Survival duration was defined as the period from the surgery to the time of death. Disease‐free survival (DFS) was defined as the period from the surgery to the time of recurrence or death. The overall survival (OS) and DFS rates were compared using the Kaplan–Meier method. Statistical significance was defined as *P* < 0.05. Statistical analysis was conducted using SPSS version 23.

## Results

### Basic characteristics

The baseline characteristics of the patients in each group are shown in Table 1. The mean age was 60.76 ± 9.90 years in the open group and 64.31 ± 9.53 years in the VATS group. The patients were predominately male: 43 patients (72.9%) in the open group and 18 (69.2%) in the VATS group. Adjacent structural invasion, including the chest wall and mediastinal structures, was significantly higher in the open group than in the VATS group (27.1% [16/59] vs. 7.7% [2/26]; *P* = 0.043). The number of cases with invasion of the chest wall was also significantly higher in the open group than in the VATS group (18.6% [11/59] vs. 0% [0/26]; *P* = 0.016). Invasion of mediastinal structures, including large vessels, was reported in 5 (8.5%) patients in the open group and 2 (7.7%) patients in the VATS group (*P* = 1.00). There were no significant differences between the groups with regard to other baseline characteristics. Additionally, there were no statistically significant differences in terms of primary cancer location, histology, or clinical stage.

**Table 1:** Baseline and preoperative characteristics

Characteristics	Open (*n* = 59)	VATS (*n* = 26)	*P*
Age	60.76 ± 9.90	64.31 ± 9.53	0.125
Gender (male)	43 (72.9%)	18 (69.2%)	0.730
Tuberculosis history	6 (10.2%)	4 (15.4%)	0.492
Smoking	37 (62.7%)	16 (61.5%)	0.918
Pack‐year	30.84 ± 18.63	36.13 ± 28.94	0.510
ECOG PS			0.092
0	53 (89.8%)	26 (100%)	
1	6 (10.2%)	0	
FVC (%)	88.04 ± 15.73	86.36 ± 21.53	0.696
FEV1 (%)	90.87 ± 19.03	91.27 ± 13.54	0.914
Tumor size (cm)	4.24 ± 1.90	3.63 ± 1.56	0.164
Invasion to other structures	16 (27.1%)	2 (7.7%)	0.043*
Radiation dose (cGy)	4942.80 ± 774.32	4757.50 ± 893.31	0.364
Location			0.050
RUL	14 (23.7%)	11 (42.3%)	
RML	1 (1.7%)	2 (7.7%)	
RLL	10 (16.9%)	7 (26.9%)	
LUL	23 (39.0%)	3 (11.5%)	
LLL	8 (13.6%)	2 (7.7%)	
> 2 lobes	3 (5.1%)	1 (3.8%)	

ECOG PS, Eastern Cooperative Oncology Group performance status; FEV1, forced expiratory volume after 1 second; FVC, forced vital capacity; LLL, left lower lobe; LUL, left upper lobe; NSCLC, non‐small cell lung cancer; RLL, right lower lobe; RML, right middle lobe; RUL, right upper lobe; VATS, video‐assisted thoracoscopic surgery.

* , statistically significant.

### Operative outcomes

Operative outcomes are shown in Table 2. VATS was converted to thoracotomy in five patients because of non‐oncologic causes (83.3%) and in one patient because of an oncologic cause (16.7%). The non‐oncologic causes included four cases of bleeding from the pulmonary artery (PA) and one case of severe adhesions. The reasons for thoracotomy conversion are described in Table 2. The case with an oncologic cause involved a lymph node (LN) that invaded the pulmonary artery (PA) that required partial resection of the PA and direct repair to achieve complete resection. Lobectomy was performed in 50 (84.7%) patients in the open group and 24 (92.3%) patients in the VATS group. The number of patients who underwent surgery combined with other procedures was significantly higher in the open group than in the VATS group (28.8% [17/59] vs. 7.7% [2/26]; *P* = 0.031). In the open group, 11 (18.6%) patients underwent combined chest wall resection, 2 (3.4%) patients underwent resection of the pericardium, and 4 (6.8%) patients underwent angioplasty. In the VATs group, one patient underwent partial thymectomy because of mediastinal soft tissue invasion and one patient underwent azygos vein resection as a result of direct cancer invasion. One patient in the open group underwent PA angioplasty as a result of PA tearing without cancer invasion. The surgical duration was significantly higher in the open group than in the VATS group (203.86 ± 65.97 vs. 173.27 ± 59.87 minutes; *P* = 0.046). There were no significant differences with regard to the surgical procedure or the operative variables between the groups (Table 3).

**Table 2:** Reasons for conversion to open thoracotomy

	Gender	Age	Reason
Oncologic cause			
1	M	58	Metastatic LN on the PA; en bloc resection of LN with pulmonary artery
Non‐oncologic cause			
1	M	67	Severe adhesions
2	F	29	Rupture of PA which was previously ligated with clip
3	F	59	Bleeding from segmental PA as a result of calcified and anthracotic LN
4	M	78	Bleeding from PA as a result of fragile artery tissue
5	M	49	Ruptured PA when encircling

LN, lymph node; PA, pulmonary artery.

**Table 3:** Surgical variables and pathologic findings

Variables	Open (*n* = 59)	VATS (*n* = 26)	*P*
Procedure			0.251
Lobectomy	50 (84.7%)	24 (92.3%)	
Bilobectomy	3 (5.1%)	2 (7.7%)	
Pneumonectomy	6 (10.2%)	0	
Combined procedure	17 (28.8%)	2 (7.7%)	0.031*
Chest wall resection	11 (18.6%)	0	0.016*
Pericardiectomy	2 (3.4%)	0	1.000
Angioplasty	4 (6.8%)	0	0.308
Other mediastinal resection	0	2 (7.7%)	0.091
Pleural adhesions	30 (50.8%)	14 (53.8%)	0.799
Surgical duration (minutes)	203.86 ± 65.97	173.27 ± 59.87	0.046*
Blood loss (cc)	407.97 ± 788.90	203.19 ± 170.06	0.062
R0 resection	58 (98.3%)	26 (100%)	0.504
Number of LNs dissected	25.32 ± 9.03	26.45 ± 10.48	0.648
Histology			0.417
Squamous cell carcinoma	31 (52.5%)	10 (38.5%)	
Adenocarcinoma	25 (42.4%)	15 (57.7%)	
Others	3 (5.1%)	1 (3.8%)	
ypT stage			0.672
T0~Tis	13 (22.0%)	7 (26.9%)	
T1	27 (45.8%)	8 (30.8%)	
T2	11 (18.6%)	7 (26.9%)	
T3	7 (11.9%)	4 (15.4%)	
T4	1 (1.7%)	0	
ypN stage			0.285
N0	32 (54.2%)	11 (42.3%)	
N1	8 (13.6%)	2 (7.7%)	
N2	19 (32.2%)	13 (50.0%)	

LN, lymph node; VATS, video‐assisted thoracoscopic surgery.

* , statistically significant.

There was no significant difference in the number of dissected LNs between the groups (open 25.32 ± 9.03 vs. VATS 26.45 ± 10.48; *P* = 0.648). Complete tumor resection was not achieved in one patient in the open group (R1 resection status), but the rate of R0 resection was not significantly different between the groups (open 98.3% [58/59] vs. VATS 100% [26/26]; *P* = 0.504). In the patient in whom complete resection was not achieved, primary cancer had invaded the PA; although partial resection and repair of the vessel were performed, the final pathologic report showed cancer invasion in the vascular resection margin. There were no significant differences in the other pathologic outcomes, including histology and pathologic stage.

### Postoperative outcomes

Postoperative outcomes, including the rate of intensive care unit stay, duration of chest tube drainage, complications, and postoperative mortality, were not significantly different between the groups (Table 4). The only significant difference was in the length of the hospital stay, which was 14.46 ± 16.94 days in the open group and 8.62 ± 4.72 days in the VATS group (*P* = 0.017). The complication rate was 28.8% in the open group and 26.9% in the VATS group (*P* = 0.858). The majority of complications were respiratory, such as pneumonia and acute respiratory distress syndrome (Table 4). Respiratory complications were reported in nine (15.3%) patients in the open group and four (15.4%) patients in the VATS group (*P* = 0.988). The incidence of BPF was higher in the open group, but there was no significant difference between them (open 4 [6.8%] vs. VATS 1 [3.8%]; *P* = 0.596). Three patients in the open group and three in the VATS group died (*P* = 0.284). All six of these patients had severe respiratory complications (pneumonia and acute respiratory distress syndrome) from which they did not recover.

**Table 4:** Postoperative outcomes

Outcome	Open (*n* = 59)	VATS (*n* = 26)	*P*
Admission to the ICU	4 (6.8%)	1 (3.8%)	0.596
Duration of chest tube drainage (days)	7.81 ± 11.36	6.92 ± 6.47	0.710
Length of hospital stay (days)	14.46 ± 16.94	8.62 ± 4.72	0.017†
Complications	17 (28.8%)	7 (26.9%)	0.858
Bleeding	1 (1.7%)	0	0.504
Prolonged air leakage	4 (6.8%)	2 (7.7%)	0.880
Pneumonia/ARDS	9 (15.3%)	4 (15.4%)	0.988
BPF	4 (6.8%)	1 (3.8%)	0.596
Operative mortality	3 (5.1%)	3 (11.5%)	0.284
Recurrence	9 (15.3%)	2 (7.7%)	0.339
Locoregional	4 (6.8%)†	0	0.174
Distant	6 (10.2%)†	2 (7.7%)	0.719

† One patient in the open group had locoregional and distant recurrence. ARDS, acute respiratory distress syndrome; BPF, bronchopleural fistula; ICU, intensive care unit; VATS, video‐assisted thoracoscopic surgery.

### Prognosis

**Figure 2:**
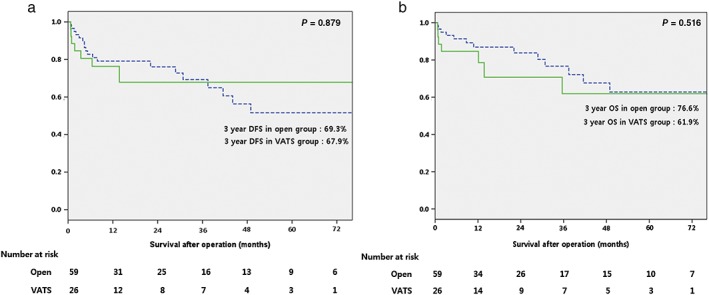
(**a**) Disease‐free survival (DFS) and (**b**) overall survival (OS). VATS, video‐assisted thoracoscopic surgery. (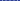), open; (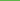), VATS.

The median follow‐up duration was 12.6 (range: 0–108) months in all enrolled patients. The recurrence rate was 12.9% (11/85) in all patients: 15.3% (9/59) in the open group and 7.7% (2/26) in the VATS group (*P* = 0.339). In the open group, three patients (5.1%) had locoregional recurrence in the lung (*n* = 1) and mediastinum (*n* = 2). Five patients (8.5%) had distant metastases in the liver (*n* = 2), brain (*n* = 2), and bone (*n* = 1). One patient (1.7%) had both lung and brain metastases. In the VATS group, two patients had distant metastases, but no locoregional recurrence; one patient had metastasis in the brain, and the other had metastases in the bone and adrenal gland. The median recurrence‐free interval was 4.4 (range: 2.1–44.0) months in the open group and 4.9 (range: 3.4–6.4) months in the VATS group (*P* = 0.687) (Table 3). The three‐year DFS (open 69.3% vs. VATS 67.9%; *P* = 0.879) and OS rates (open 76.6% vs. VATS 61.9%; *P* = 0.516) were not significant in either group (Fig 2).

## Discussion

VATS anatomic lung resection is considered the standard treatment for early‐stage NSCLC, but in advanced lung cancer patients who have received neoadjuvant CRT, the feasibility and safety of pulmonary resection under VATS is still unclear. The technical difficulties associated with VATS pulmonary resection after neoadjuvant therapy result from adhesion formation, tissue fragility, and decreased healing.^8^ Moreover, radiation therapy leads to the development of fibrosis, whereby collagen deposition by fibroblasts into the interstitium leads to chest tissue edema or adhesions, thus increasing the difficulty of surgery.^3^, ^9^

In our study, the operative outcomes of the VATS group were comparable to the open group, suggesting that VATS pulmonary resection after neoadjuvant CRT is feasible and safe. In six patients, VATS was converted to thoracotomy, with a conversion rate of 18.8% (6/32). Only one patient (3.1%) was converted because of an oncologic cause, which was the result of a metastatic LN on the PA that was resected en bloc with the PA. The five non‐oncological causes (15.6%) were severe adhesions in one patient and control of bleeding from the PA in the other four. The reasons for each case are as follows: the previous PA ligated with the clip site had ruptured; bleeding from the segmental PA as a result of a calcified and anthracotic LN; bleeding from the PA because of fragile PA tissue (in a 78‐year‐old man); and a rupture of the PA when it was encircled. These findings suggest that neoadjuvant CRT did not increase the incidence of thoracotomy conversion during VATS. Our results are similar to those reported in other studies. The rate of unexpected conversion to thoracotomy in lung cancer patients without CRT is reported to range from approximately 2.5% to 23.0%.^10^ In a previous study, the reasons for conversion included severe fibrocalcific LNs and uncontrolled bleeding; only 15.9% of patients were converted to thoracotomy because of oncological causes. A different study, in which patients underwent VATS lobectomy after induction therapy, reported a conversion rate of 12.5%.^2^ In the present study, more complex procedures were performed in the open group than in the VATS group (28.8% [17/59] vs. 7.7% [2/26]; *P* = 0.031) because these patients had more extensive invasion of the adjacent structures compared to the VATS group (27.1% [16/59] vs. 7.7% [2/26]; *P* = 0.043). As a result, the surgical duration was significantly longer in the open group (*P* = 0.046).

Induction CRT appeared to increase the risk of postoperative complications by causing changes that led to increased tissue fragility, delayed tissue healing, and fibrotic changes in the pulmonary tissues. In previous studies, the incidence of postoperative respiratory complications in neoadjuvant chemotherapy (not chemoradiotherapy) patients was 3–4.3%, and the incidence of BPF was 0.7–2%.^6^, ^8^ In our study, pneumonia occurred in 15% of patients, and 5.8% patients developed a BPF. This was slightly higher in the neoadjuvant CT group in the previous study, but could be the effect of radiation therapy, which increases tissue fragility and delays tissue healing. However, our results showed that there was no difference in the complication rate according to the surgical approach. The rate of complications –including BPF, bleeding, and respiratory complications – was not significantly different between the groups (open 28.8% vs. VATS 26.9%; *P* = 0.858). The operative mortality was 7.06% (6/85), which included three patients from each group; the reason for mortality was respiratory failure.

With regard to oncologic outcomes, complete resection and recurrence rates were comparable between the groups. In one patient, VATS was converted to thoracotomy and complete resection was not achieved. In this patient, primary cancer had invaded the PA, thus partial resection and repair of the vessel was performed; the final pathologic report showed cancer invasion in the vascular resection margin. Eleven patients experienced recurrence, including two patients from the VATS group. These two patients had distant recurrences, but no local recurrence, suggesting that they had undergone sufficient surgical resection, including mediastinal node dissection. However, in the open group, there were five cases of locoregional recurrence (3 patients had metastases to the lung, 2 had metastases to the mediastinum). Although these patients underwent complete resection, these recurrences could be attributed to the greater frequency of adjacent structure involvement.

This study has some limitations. First, the study was a retrospective single‐center design. Second, the number of patients who underwent VATS resection was relatively small and the follow‐up period was short. Finally, the selection of the surgical procedure performed was based on the surgeon's preference. Additional randomized controlled studies with larger study groups and longer follow‐up periods are needed to overcome the limitations of this study.

In conclusion, VATS pulmonary resection following neoadjuvant CRT in selected patients shows results comparable to those of conventional thoracotomy in terms of postoperative outcomes, operative morbidities, and survival rate. These findings suggest that VATS resection could be safely and effectively performed after neoadjuvant CRT in selected advanced‐stage NSCLC patients who do not require massive resection of adjacent structures.

## Disclosure

No authors report any conflict of interest.
